# Tumor-associated macrophages in direct contact with prostate cancer cells promote malignant proliferation and metastasis through NOTCH1 pathway

**DOI:** 10.7150/ijbs.73141

**Published:** 2022-10-03

**Authors:** Fei Shi, Meng-Hao Sun, Zheng Zhou, Lei Wu, Zheng Zhu, Shu-Jie Xia, Bang-Min Han, Yu-Yang Zhao, Yi-Feng Jing, Di Cui

**Affiliations:** 1 Department of Urology, Shanghai General Hospital Affiliated to Shanghai Jiao Tong University, School of Medicine, Shanghai, China.; 2 Institute of Urology, Shanghai Jiao Tong University, Shanghai 200080, China.; 3 Department of Urology, Shanghai General Hospital, Nanjing Medical University, Shanghai 200080, China.; 4 Department of Internal Medicine, Division of Hematology/Oncology, University of California Davis, Sacramento, CA 95817, USA.

**Keywords:** prostate cancer (PCa), tumor-associated macrophage (TAM), NOTCH1 signaling, MAML2, cancer progression

## Abstract

**Background:** M2 macrophages are well accepted to promote cancer progression in the prostate cancer (PCa). Paracrine is the principally studied mode of communication between M2 macrophages and tumor cells. In addition to this, we present here a novel model to demonstrate these cellular communications.

**Methods:** PCa cells were co-cultured with THP-1/ human peripheral blood mononuclear cells derived M2 macrophages in direct contact manner. Cancer cell proliferation and invasion were examined to explain how direct contact communicates. Cell-based findings were validated in two xenograft models and patients samples.

**Results:** M2 macrophage direct contact induced a higher proliferation and invasion in PCa cells when compared with noncontact coculture manner. In direct contact manner, NOTCH1 pathway was greatly activated in PCa cells, induced by elevated γ-secretase activity and higher coactivator MAML2 expression. Additionally, blocking γ-secretase activity and depletion of MAML2 completely abolished M2 macrophage direct contact-mediated PCa cell proliferation and invasion. *In vivo*, inhibiting NOTCH1 signalling impaired M2 macrophage-mediated PCa tumor growth and lung metastasis. Notably, M2 macrophage infiltration as well as high NOTCH1 signaling in cancer cells indicated more aggressive features and worse survival in PCa patients.

**Conclusion:** Our results demonstrated the cell-cell direct contact pattern is an important way in PCa microenvironment cell communication. In this manner, elevated γ-secretase activity and MAML2 expression induced higher NOTCH1 signalling in PCa cells, which increased tumor cells proliferation and invasion. This potentially provided a therapeutic target for PCa.

## Introduction

Prostate cancer (PCa) is one of the leading causes of cancer mortality worldwide. During cancer progression, inflammation in the tumor microenvironment (TME) highly promotes metastasis and castration resistance 1-3. Macrophages are the functionally plastic leukocytes in the tumor inflammatory microenvironment. Traditionally, inflammatory macrophages are polarized into two main phenotypes: M1 (classically activated) and M2 ones (alternatively activated, M2a-d). But nowadays, tumour-associated macrophages (TAMs) are more considered as a characterized population with both M1 and M2 marker genes expression. Very recently, macrophage single cell sequence confirmed this in prostate tumor tissues, though they favour more signature M2 makers expression than M1 4-6. Despite its limitation and oversimplification, M1/M2 polarization is still widely used in TAMs research before better category labels 7.

In PCa, M2 macrophages are well accepted as the main ones to promote tumor progression and drug resistance in PCa 8, 9.To date, paracrine signaling is the most studied mechanism, as M2 macrophages produce a variety of growth factors, chemokines, exosomes and amino acids. However, targeting M2 macrophages paracrine was not sufficient to impair tumor progression 10. This means that paracrine mechanism is far less enough to form the complex of the TME niche. Thus, a comprehensive understanding of the mechanism of M2 macrophage-mediated cancer malignancy is clinically beneficial for developing new therapeutic strategies to delay prostate cancer progression.

The NOTCH signaling pathway is a ligand-receptor binding-dependent pathway. In brief, NOTCH receptors (NOTCH1-4) bind to ligands and then release their intracellular domain (NICD) through γ-secretase cleavage. NICD subsequently translocates to the nucleus and forms a transcriptional complex with the coactivator Mastermind-like (MAML1,2,3) and DNA-binding protein CSL 11-13. This complex promotes transcriptional activation of NOTCH target genes, including HEY1 and HES1 14. Increasing evidence indicates that NOTCH signaling is an oncogenesis pathway that promotes cell proliferation, stem cell phenotype and angiogenesis in PCa 15, 16. Moreover, the NOTCH signaling pathway also regulates epithelial-mesenchymal transition (EMT) 17.

In this study, we disclose a cell-cell contact model of M2 macrophages and PCa cells. Compared with a paracrine manner, M2 macrophages promoted higher PCa cell proliferation and metastasis in a direct contact manner. Mechanistically, M2 macrophages directly increase γ-secretase activity and MAML2 expression to activate the NOTCH signaling pathway. Moreover, increased M2 macrophages infiltration combined with high NICD expression indicates the worst clinical outputs. Our findings reveal a novel model of PCa cell proliferation and metastasis through direct M2 macrophages-mediated cell contact.

## Materials and Methods

### Patient and tissue samples

This study was approved by the ethics committee of Shanghai General Hospital (Shanghai, China). PCa tissue samples and pathology data from 89 patients were collected from Shanghai General Hospital. These patient tissues were obtained after prostatectomy. The clinical features were characterized by at least two professional pathologists using a double-blind method. All patients were informed and signed a consent form. Patients were followed for at least 5-7 years.

### Cell culture and reagents

DU145, 22RV1 and THP-1 cells were obtained from the Chinese Academy of Sciences Committee on Type Culture Collection Cell Bank (Shanghai, China). All cell lines were cultured in RPMI-1640 medium (Sigma-Aldrich; Merck, Darmstadt, Germany) supplemented with 10% fetal bovine serum and maintained in 5% CO_2_ at 37 °C. These cell lines were authenticated by the Chinese Academy of Sciences Committee using short tandem repeat (STR) profiling. To obtain THP-1-derived M2 macrophages, THP-1 cells (2×10^5^ cells/well) were treated with 10 ng/ml phorbol 12-myristate 13-acetate (PMA) for 24 h and then cultured with IL-4 (25 ng/ml) and IL-13 (25 ng/ml) for another 48 h. Human PBMCs (peripheral blood mononuclear cells) were purchased from Shycbio (Shanghai, China) and stored in a -80 ℃ ultralow temperature freezer. For PBMC-derived M2 macrophages, an appropriate amount of complete M2 macrophage Generation Medium DXF (Promo Cell, Heidelberg, Germany) was incubated with the cells according to the manufacturer's instructions.

### Flow cytometry

CD163 and CD206 expression in M2 macrophages was measured by flow cytometry using anti-CD163 and anti-CD206 antibodies (BD Biosciences, San Diego, CA, USA) according to the manufacturer's instructions. For THP-1 and PMBC-derived M2 macrophages, cells were dissociated using 0.25% trypsin and washed twice with cold PBS. Cells were incubated with anti-CD163 and anti-CD206 antibodies for 30 minutes, washed twice with cold PBS in the dark and then analyzed using a BD Accuri C6 flow cytometer (BD Biosciences, San Diego, CA, USA). The separated tumor cells and M2 macrophages were identified and cultured under the indicated conditions.

### Cell coculture system establishment

DU145 and 22RV1 cells were infected with lentivirus carrying the mCherry-luciferase fragment. Stable clones were picked for coculture and cell viability assays. For the direct coculture model, THP-1 or PBMC-derived M2 macrophages (1×10^5^ cells) were seeded together with mCherry-luciferase-tagged DU145 and 22RV1 (5×10^5^ cells) stable clones into 6-well plates at a ratio of 1:5 for 3 days. FACS was used to separate M2 and mCherry DU145 and 22RV1 cells for the next coculture assay. For at least 5 passages, cocultured mCherry DU145 and 22RV1 cells were separated for* in vitro* assays. For the indirect coculture model, transwell chamber was introduced to construct a non-contact environment with THP-1 or PBMC-derived M2 macrophages (1×10^5^ cells) in the upper compartment and mCherry-luciferase-tagged DU145 and 22RV1 (5×10^5^ cells) in the lower compartment. The experimental group was indicated as control (DU145 or 22RV1 cells), non-contact (DU145 or 22RV1 cells indirect coculture with M2 macrophage), contact (DU145 or 22RV1 cells direct coculture with M2 macrophage).

### Total protein extraction and western blotting

Cocultured DU145 and 22RV1 cells were lysed in RIPA buffer (NCM, Suzhou, China) containing 1% (v/v) protease inhibitor cocktail (Roche, Indianapolis, IN). The amount of protein in each sample was determined using the Pierce BCA Protein Assay (Thermo Scientific, Waltham, MA, USA). Total protein (20 µg) was separated on 10% sodium dodecyl sulfate-polyacrylamide gel electrophoresis (SDS-PAGE) gels and transferred onto polyvinylidene fluoride (PVDF) membranes (Millipore, Billerica, MA, USA). The membranes were blocked with skim milk and then incubated with primary antibodies against NOTCH1 (1:1000), N1ICD (1:1000), HEY1 (1:1000), HES1 (1:1000), E-cadherin (1:800), and N-cadherin (1:1000) at 4 °C overnight and anti-rabbit or anti-mouse HRP-conjugated secondary antibodies for two hours at room temperature. Protein bands were visualized by ECL (NCM, Suzhou, China) using the ECL Detection System (Thermo Scientific, Waltham, MA, USA).

### Immunofluorescence (IF) and Immunohistochemistry (IHC)

Formalin-fixed, paraffin-embedded tissue samples were cut into 4-μm-thick sections. Antigen retrieval was performed using a pressure cooker for 3 min in 0.01 M citrate buffer (pH 6.0). DU145 and 22RV1 cells were washed with PBS and fixed in 4% paraformaldehyde for 20 min at room temperature followed by treatment with 0.05% Triton X-100 at 4 °C for 5 min. Sections and cells were blocked with 1% BSA for 1.5 h at room temperature and incubated with primary antibodies against CD68 (1:500; Trevigen), CD206 (1:500; Abcam, London, UK), N1ICD (1:1000; Abcam, London, UK), E-cadherin (1:500; Novus, Centennial, USA) or N-cadherin (1:500; Novus, Centennial, USA) at 4 °C overnight. The samples were incubated with secondary antibodies conjugated with Alexa Fluor or HRP for 1 h at room temperature. Tissues or cells were counterstained with 4′,6-diamidino-2-phenylindole dihydrochloride (DAPI) to detect nuclei and visualized by fluorescence microscopy. For IHC, the secondary antibody was diluted to 1:750 for recognizing primary antibodies. The staining for IHC was visualized using the VECTASTAIN ABC peroxidase system and peroxidase substrate DAB kit.

### Cell bioluminescence assay

Lentivirus-infected DU145-luc and 22RV1-luc cells were directly cocultured with M2 macrophages for five passages and then seeded into 12-well plates. Fluorescein potassium (10 ng/ml) was used as the substrate and added to culture wells for 15 minutes in a dark environment. An IVIS-200 bioluminescence and fluorescence imaging system (Caliper Life Sciences Inc., Hopkinton, MA, USA) was used to detect the cell luciferase value. The luciferase value was positively corelated with tumor cell number (Figure S1A-B).

### Plasmid construction and lentiviral transfection

Plasmids carrying negative control shRNA or NOTCH1 shRNA were generated by OBiO (Shanghai, China). To knockdown NOTCH1, DU145 and 22RV1 cells (1×10^5^ per well) were seeded in 6-well plates. After they reached approximately 75% confluency, the culture medium was changed to fresh culture medium containing lentiviral particles (OBiO, Shanghai, China) with NOTCH1-shRNA or negative control using FuGENE6 (Roche, Indianapolis, IN) according to the manufacturer's instructions. After 24 h of transfection, the culture medium was replaced with fresh medium containing puromycin to select and create a stable line.

### RNA sequencing

Briefly, DU145 and 22RV1 cells were either noncontact (control group) or contact (experimental group) cocultured with THP-1-derived M2 macrophages for 5 passages. Cells were collected, and total RNA was extracted using TRIzol Reagent according to the manufacturer's instructions (Invitrogen). Following the TruSeqTM RNA sample preparation kit from Illumina (San Diego, CA), the RNA-seq transcriptome library was prepared using 10 μg of total RNA. The raw paired-end reads were trimmed and quality controlled using SeqPrep and Sickle with default parameters. Then, the clean reads were separately aligned to the reference genome with orientation mode using TopHat software. Differentially expressed genes were calculated according to the fragments per kilobase of exon per million mapped reads (FRKM) method. The quantification of gene abundance was qualified using RSEM. The R statistical package software EdgeR was utilized for differential expression analysis. In addition, functional enrichment analysis, including GO and KEGG analyses, was performed to identify which differentially expressed genes mapped to significantly enriched GO terms.

### Animal experiment

Animal studies were performed according to the US Public Health Service Policy on Humane Care and Use of Laboratory Animals. The animal study protocol was approved by the Scientific Investigation Board of Shanghai General Hospital. Briefly, a total of 1 × 10^6^ DU145-luc and 22RV1-luc cells from each clone were suspended in sterile PBS, mixed with 2×10^5^ THP-1-derived M2 and then injected subcutaneously into male nude mice (4-6 weeks old). An IVIS-200 bioluminescence and fluorescence imaging system (Caliper Life Sciences Inc., Hopkinton, MA, USA) was used to dynamically measure the growth of tumors. Tumor xenograft size was recorded every 7 days using the following formula: V (mm^3^) = width^2^ (mm^2^) × length (mm)/2. After 6 weeks, all mice were sacrificed, and the xenografts developed from each mouse were resected and evaluated for various parameters, including tumor incidence, size, weight, and immunostaining, at the indicated time points. Tail vein injection of luciferase-labeled DU145 and 22RV1 cells, mixed with 2×10^5^ THP-1-derived M2 macrophage was also performed on 4- to 6-week-old male nude mice after direct contact with M2 macrophages. Seven weeks after the initial injection, an IVIS-200 bioluminescence and fluorescence imaging system (Caliper Life Sciences Inc., Hopkinton, MA, USA) was used to measure the tumor volume of each treatment group. After 8 weeks, all mice were sacrificed, and necropsies were performed. Various parameters, including the number, weight, and location of individual tumor nodules, were evaluated and then processed for histological assessment.

### Statistical analysis

All experiments were performed in triplicate. The data are presented as the mean ± SEM. Statistical analysis was performed using either the chi-square test, Cox regression, or unpaired t test and ANOVA. Differences in survival were analyzed by Kaplan-Meier curves using the log-rank test. P < 0.05 was considered statistically significant.

## Results

### M2 macrophage direct contact promotes PCa cell proliferation and invasion

**Figure 1:**
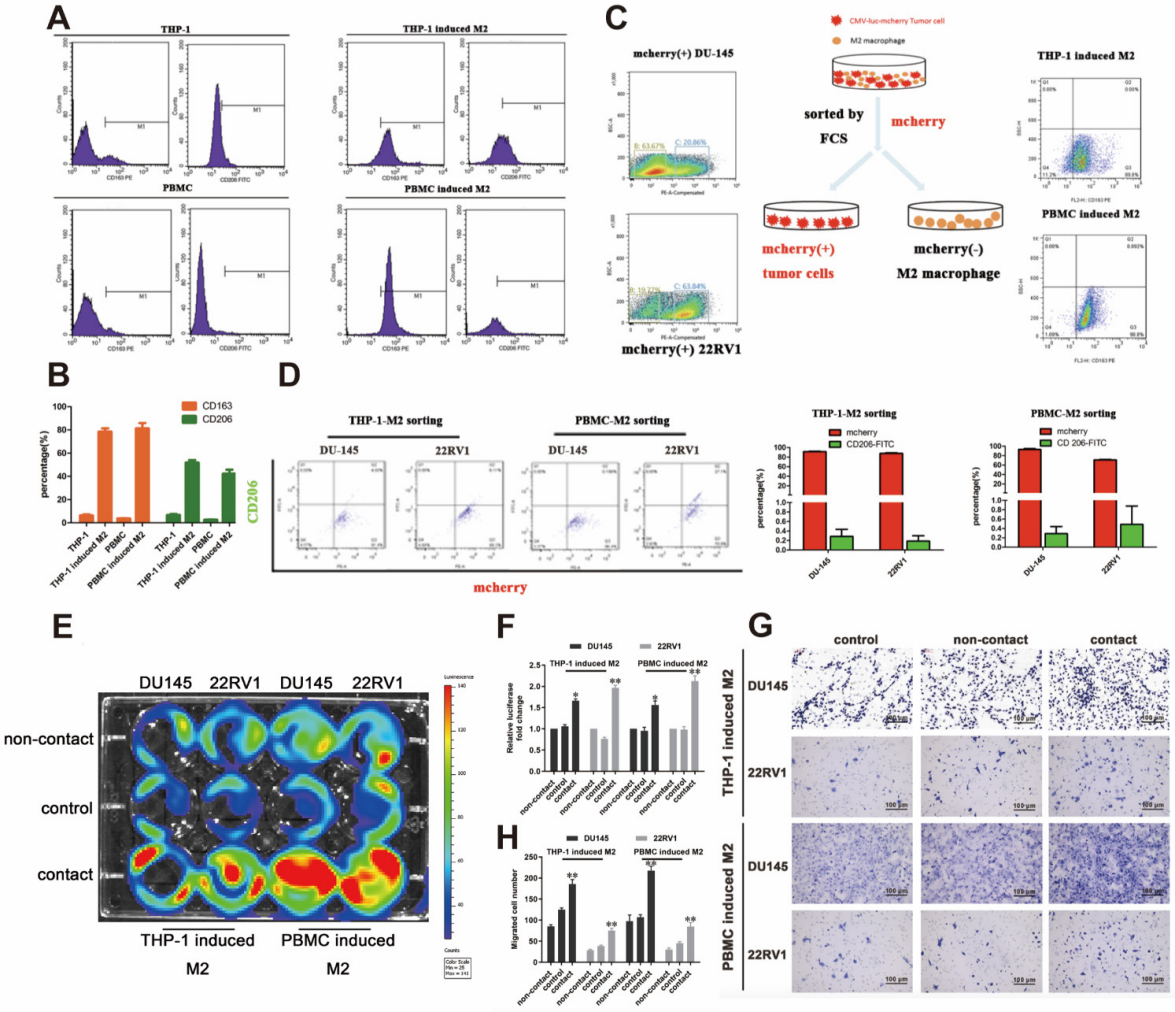
** M2 macrophage promote PCa cell proliferation and invasion through direct contact. (A, B)** Flow cytometry (FCM) detection of THP-1 and PBMC-induced M2 macrophages makers. **(C)** The strategy and schematic diagram for direct coculture and separation of TAMs and mCherry-luciferase-labeled DU145 and 22RV1 cells. FACS was used to separate tumor cells and TAMs according to the color channel. **(D)** FCM identified the separated tumor cells by detecting mCherry and CD206. **(E)** Bioluminescence assay of DU145-luc and 22RV1-luc. TAMs (THP-1 cells and PBMCs derived) in direct contact increased the luminescence values of DU145-luc and 22RV1-luc cells compared with those of the noncontact and control groups (left). **(F)** Quantification data of the cell luminescence value (right). **(G)** Matrigel-based Transwell invasion assay of DU145-luc and 22RV1-luc cells. TAMs (THP-1 and PBMC-derived) in direct contact increased the invasive ability of DU145-luc and 22RV1-luc cells compared with the noncontact and control groups. **(H)** The quantification data of invasive assay (right) (*p<0.05, **p<0.01).

We established a cell direct contact model of M2 macrophages and PCa cells as indicated by the methods. THP-1- or PBMC-derived M2 macrophages were identified by CD163 and CD206 expression (Figure 1A, 1B). mCherry-luciferase-tagged DU145 and 22RV1 (named DU145-luc and 22RV1-luc) cells were directly cocultured with M2 macrophages for three days as a cycle. FACS showed no change in mCherry intensity or CD206 expression after cell digestion (Figure 1C, 1D). After five growth cycles, the luciferase activity showed that direct contact coculture significantly induced higher prostate cancer proliferation than noncontact coculture and the control group (Figure 1E, 1F). Further, we investigated the variation in PCa metastasis ability after direct contact with M2 macrophages. Similar, direct contact coculture facilitated more prostate cancer cells invading into Matrigel compared with the other two groups (Figure 1G, 1H). In conclusion, these data revealed that M2 macrophage promoted PCa cell proliferation and invasion through direct contact manner.

### M2 macrophages direct contact activates the NOTCH1 signaling pathway in PCa cells

**Figure 2:**
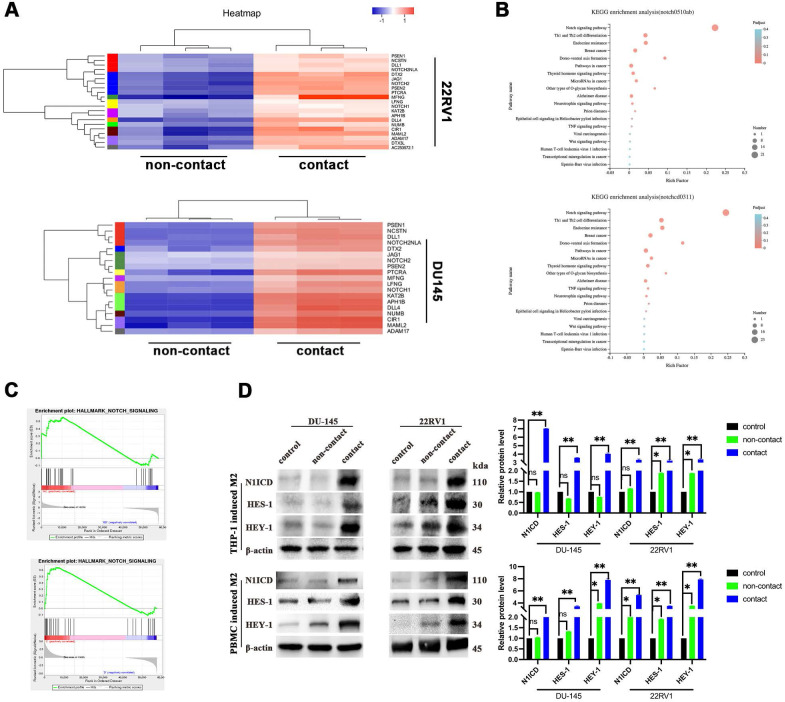
** M2 macrophages contact activates NOTCH signaling in PCa cells. (A-C)** RNA-sequence data of M2 macrophage contact (experimental group) and noncontact (control group). A, Gene cluster analysis of DU145-luc and 22RV1-luc. B, KEGG enrichment analysis of DU145-luc and 22RV1-luc showed that M2 macrophage direct contact increased NOTCH signaling pathway enrichment. C, GSEA showed that direct M2 macrophage contact promoted NOTCH-related gene enrichment. **(D)** Western blot assay of NOTCH signaling-related protein expression. M2 macrophages (THP-1 and PBMC-derived) in direct contact increased N1ICD, HES1, and HEY1 expression in DU145-luc and 22RV1-luc cells compared with that in the noncontact and control groups.

To explore the mechanism, we applied RNA sequencing of PCa cells after direct contact coculture with THP-1-derived M2 macrophages. Gene cluster analysis is presented as a heatmap (Figure 2A). NOTCH signaling-related genes were significantly enriched after M2 macrophage direct contact, as shown by KEGG pathway analysis and GSEA (Figure 2B, 2C). The NOTCH1 intracellular domain N1ICD and NOTCH1 signaling target genes HES1 and HEY1 were all increased after contact with M2 macrophages (Figure 2D).

**Figure 3:**
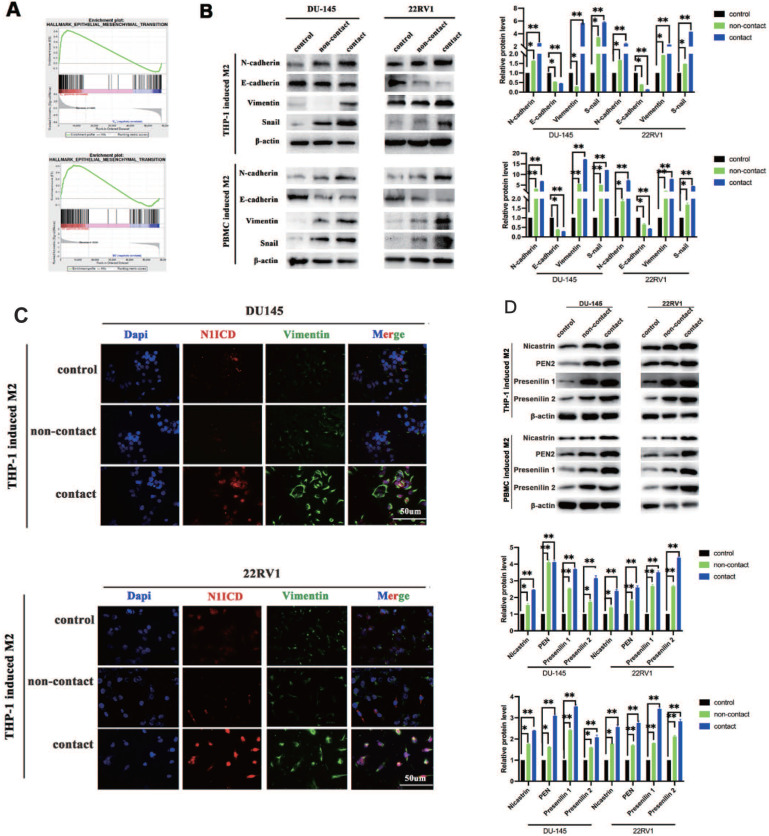
** M2 macrophage contact activates EMT signaling in PCa cells. (A)** GSEA showed that M2 macrophages direct contact promoted EMT-related gene enrichment in DU145-luc and 22RV1-luc cells. **(B)** Western blot assay of EMT signaling-related protein expression. TAMs (THP-1 and PBMC-derived) directly contacted increased N-cadherin, vimentin, and Snail and decreased E-cadherin expression in DU145-luc and 22RV1-luc cells compared with the noncontact and control groups. **(C)** Immunofluorescence assays showed that TAMs in direct contact increased N1ICD (far red) and vimentin (green) expression of DU145-luc and 22RV1-luc compared with the noncontact and control groups. (Scale bar=50 µm). **(D)** Western blot assay detected γ-secretase component protein expression. M2 macrophages (THP-1 and PBMC-derived) directly contact increases Nicastrin, PEN2, Presenilin1 and Presenilin2 expression in DU145-luc and 22RV1-luc cells compared with the noncontact and control groups.

Epithelial-mesenchymal transition (EMT) is the core step for tumor metastasis, and NOTCH signaling regulates EMT in cancers 18. As expected, RNA sequencing data showed that EMT-associated genes were significantly enriched in DU145 and 22RV1 cells after direct contact with M2 macrophages (Figure 3A). Furthermore, M2 macrophage direct contact increased N-cadherin, Snail, and vimentin expression and decreased E-cadherin expression in PCa cells, which induced the PCa EMT process (Figure 3B). Moreover, the activation of EMT was accompanied by NOTCH1 signaling activation after direct contact from increased Vimentin and N1ICD co-expression (Figure 3C). These data indicated that M2 macrophages direct contact promotes PCa metastasis by activating NOTCH1 pathway and EMT.

### M2 macrophages direct contact activates the PCa NOTCH1 pathway by increasing γ-secretase activity

To further explore the key step of NOTCH signaling activation, we detected NOTCH signaling ligands in M2 macrophages and receptors in PCa cells in direct contact coculture. No expression change was found in either ligands (Dll1, Dll3, Dll4 and Jag1, Jag2) or receptors (NOTCH1,2,3,4) (Figure S2). γ-Secretase components are crucial for NOTCH receptor cleavage to activate the intercellular domain. We found that γ-secretase component (Nicastrin, PEN2, Presenilin1 and Presenilin2) expression was increased in M2 macrophage direct-contactd PCa cells (Figure 3D). Although recent studies demonstrated that AR signaling was involved in NOTCH signaling activation 19, GSEA results did not indicate that PCa cell AR signaling was significantly changed in a direct contact manner (Figure S3A). In addition, AR and PSA protein expression was not altered after direct contact (Figure S3B). These data imply that M2 macrophage direct contact activates PCa NOTCH1 signaling by upregulating γ-secretase in PCa cells.

### Inhibiting NOTCH1 singaling impaired M2 macrophage direct contact-induced cell proliferation and invasion

**Figure 4:**
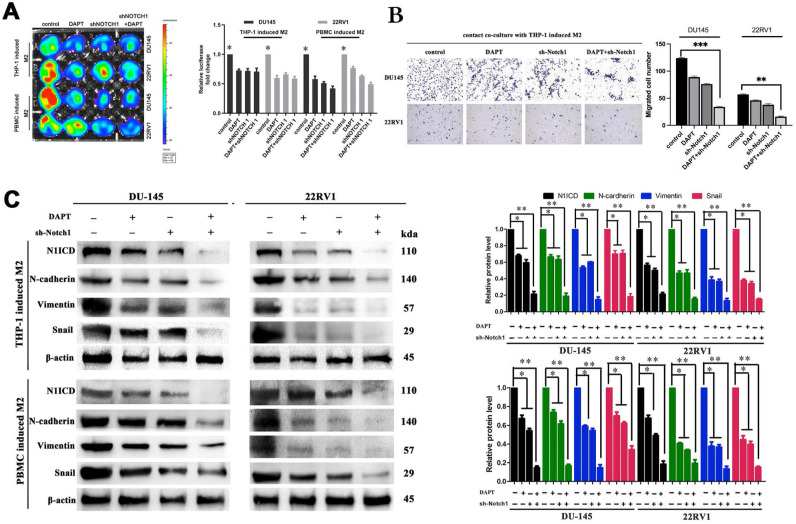
** Inhibiting NOTCH1 singaling impaired M2 macrophages-mediated cell proliferation and invasion. (A)** Bioluminescence assays showed that inhibiting NOTCH signalling impaired M2 macrophage direct contact-mediated DU145-luc and 22RV1-luc cell proliferation. **(B)** Matrigel-based Transwell invasion assays showed that γ-secretase inhibition in NOTCH1-depleted DU145-luc and 22RV1-luc cells abolished M2 macrophages direct contact-mediated cell invasive ability. **(C)** Western blot assay showed that inhibiting γ-secretase activity significantly reduced N1ICD, N-cadherin, vimentin and Snail expression in NOTCH1-depleted DU145-luc and 22RV1-luc cells (*p<0.05, **p<0.01).

Inhibiting the NOTCH1 pathway with shNOTCH1 and the γ-secretase inhibitor DAPT significantly reduced DU145-luc and 22RV1-luc cell proliferation ability in an M2 macrophage direct-contact manner (Figure 4A), as well as invasion (Figure 4B). The EMT process of PCa was also impaired after treatment, with decreased N1ICD, N-cadherin, vimentin and Snail expression (Figure 4C). However, without coculture with M2 macrophage either DAPT or knockdown Notch1 had little influence on EMT markers (E-cadherin, N-cadherin, vimentin and Snail) (Figure S4). Together, we revealed that M2 macrophage direct contact promoted cell proliferation and invasion, which partially relied on γ-secretase proteolytic cleavage activity in the NOTCH1 pathway.

### M2 macrophage direct contact also activates the PCa NOTCH1 pathway through elevated coactivator MAML2

**Figure 5:**
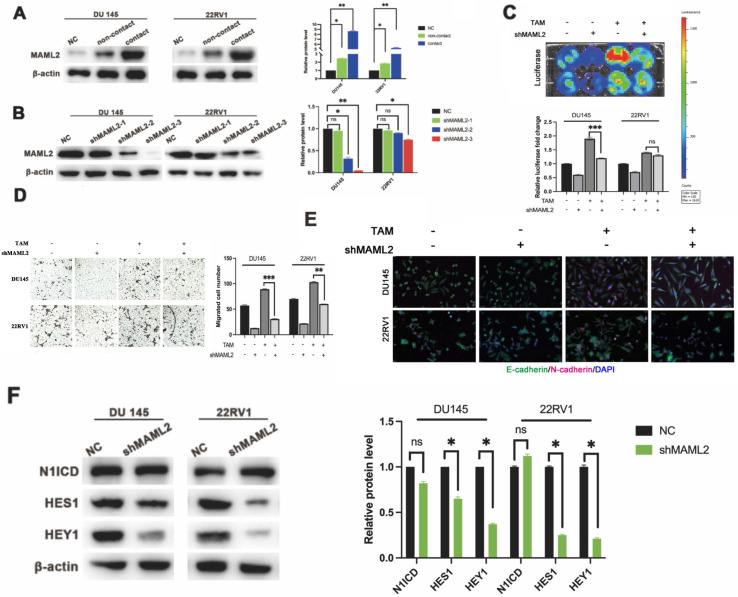
** MAML2 is essential for TAM-mediated NOTCH1-dependent transcription. (A)** Western blot assay showed that M2 macrophages (THP-1 and PBMC-derived) in direct contact increased MAML2 expression in DU145-luc and 22RV1-luc cells compared with the noncontact and control groups. **(B)** Lentivirus containing the shMAML2 fragment was infected into DU145 and 22RV1 cells, and Western blotting was used to detect the knockdown effect. **(C)** Bioluminescence assays showed that MAML2 depletion in DU145 and 22RV1 cells abolished M2 macrophages direct contact-mediated cell proliferation. **(D)** Matrigel-based invasion assays showed that MAML2 depletion in DU145 and 22RV1 cells abolished M2 macrophages direct contact-mediated cell invasion. **(E)** Immunofluorescence assays showed that MAML2 depletion inhibited M2 macrophages direct contact-induced EMT by decreasing N-cadherin (far-red) expression in DU145 and 22RV1 cells. **(F)** Western blot assay showed that knocking down MAML2 did not alter M2 macrophages-mediated N1ICD release but reduced HES1 and HEY1 expression (scale bar=50 µm, *p<0.05, **p<0.01).

*In vitro* data showed that neither γ-secretase inhibitor nor shNOTCH1 was enough to diminish PCa malignant progression in a direct contact manner. There might exist a supplemental mechanism in addition to γ-secretase upregulation. MAML2 is a coactivator in the NOTCH1 pathway that binds to NICD to form transcriptionally activating complexes 12, 20. We observed that MAML2 was increased in DU145-luc and 22RV1-luc cells in direct contact with M2 macrophages (Figure 5A). To investigate the role of MAML2 in the contact coculture phenotype, we first established transfected lentivirus carrying shMAML2 PCa cell lines (Figure 5B). MAML2 knockdown reduced PCa cell proliferation and invasion in a contact coculture manner (Figure 5C, D). Moreover, MAML2 knockdown also reduced M2 macrophage contact-induced PCa EMT, upregulation of E-cadherin and downregulation of N-cadherin (Figure 5E). Interestingly, knocking down MAML2 did not alter M2 macrophage contact-mediated N1ICD release but reduced the expression of the NOTCH1-targeting genes HES1 and HEY1 (Figure 5F). Together, these data implied that upregulation of MAML2 is another way to explain M2 macrophage contact-mediated NOTCH1 pathway activity.

### M2 macrophage depletion and NOTCH1 signaling blockade prohibited PCa cell proliferation and metastasis *in vivo*

**Figure 6:**
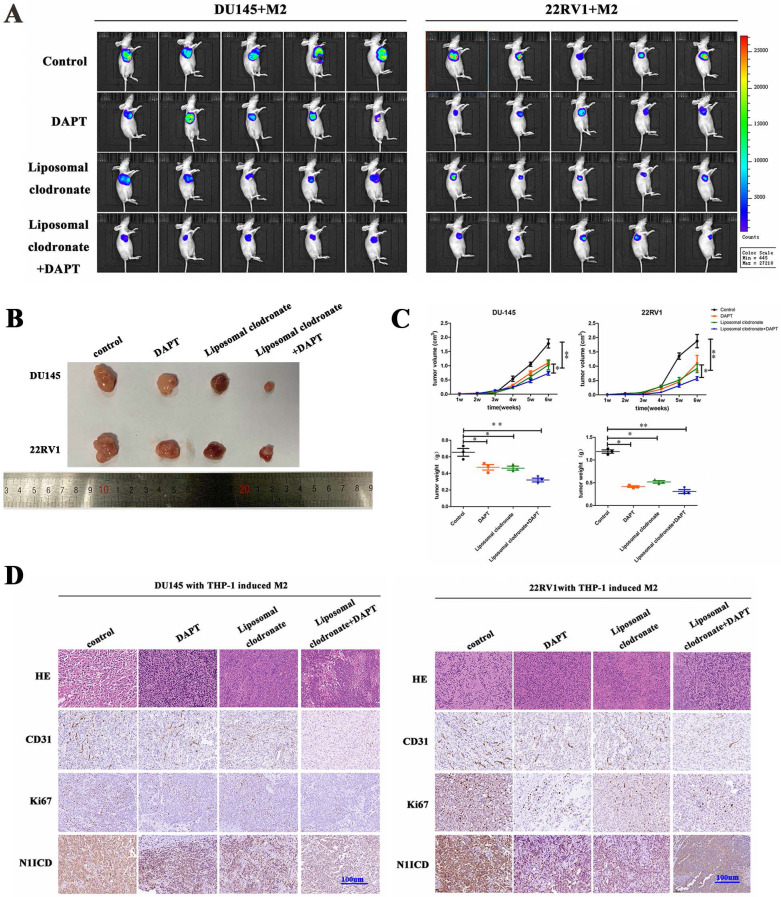
** M2 macrophages depletion and NOTCH1 signaling inhibition impaired PCa cell-derived xenograft growth. (A)** Xenografts (n=5) were obtained from nude mice that were injected with 1×10^6^ DU145-luc and 22RV1-luc cells (mixed with 2×10^5^ THP-1-derived M2 macrophages), and pharm treatment was used (including PBS, DAPT, liposomal clodronate or combination) until tumor xenografts were formed. An IVIS-200 bioluminescence system was used to monitor tumor growth once a week, and node mice were sacrificed after 4 weeks. **(B)** Tumor xenografts were displayed as indicated. **(C)** The curve of tumor volume and weight of each group (n=5) was analyzed; group differences were analyzed via ANOVA. **(D)** Histological staining of CD31, Ki67 and N1ICD is displayed as indicated (*p<0.05, **p<0.01).

*In vivo*, we obtained DU145-luc- and 22RV1-luc-derived xenografts by injecting tumor cells into mouse axilla. Liposomal clodronate and DAPT were intraperitoneally injected into the experimental group. Tumor growth curves demonstrated that tumor growth was slightly inhibited after either liposomal clodronate or DAPT treatment. Furthermore, when liposomal clodronate and DAPT were combined, tumor growth was significantly inhibited at extremely low levels (Figure 6A, B). The volume and weight of xenografts were also identical in this result (Figure 6C). Tumor Ki67 expression was also significantly reduced in the liposomal clodronate and DAPT combined treatment group (Figure 6D).

**Figure 7:**
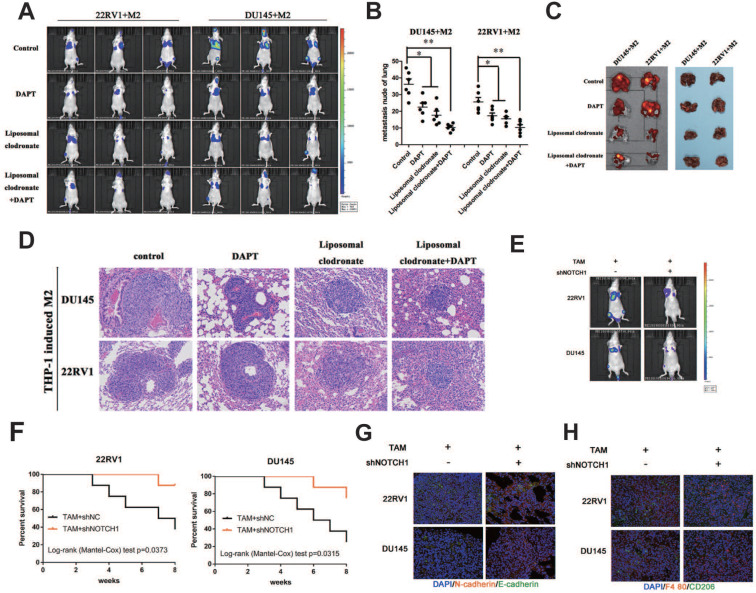
** M2 macrophages depletion and NOTCH1 signaling inhibition impaired PCa cell lung metastasis. (A)** A lung metastasis model (n=3) was established by tail injection of 1×10^6^ DU145-luc and 22RV1-luc cells (mixed with 2×10^5^ THP-1-derived TAMs) into nude mice. Pharm treatment was used (including PBS, DAPT, liposomal clodronate or combination) once tail injection was finished. An IVIS-200 bioluminescence system was used to monitor lung metastasis once a week, and node mice were sacrificed after 6 weeks. **(B)** Quantification of lung metastasis nodes is displayed as indicated. **(C)** Metastatic lungs are displayed as indicated (left, fluorescence; right, brightness, n=3). **(D)** Alizarin red staining was used to observe the metastatic node of each group. **(E)** DU145-luc and 22RV1-luc cells (mixed with 2×10^5^ THP-1-derived M2 macrophages) (1×10^6^) were used to establish a lung metastasis model (n=3). Adenovirus carrying NOTCH1 shRNA was injected into mouse lung tissue until tumor metastasis formation. An IVIS-200 bioluminescence system was used to monitor lung metastasis diminishing. **(F)** Kaplan-Meier survival curves are displayed as indicated. **(G)** Intralung injection of shNOTCH1 adenovirus suppressed metastatic MET-associated tumor cell plantation (N-cadherin, far-red; E-cadherin, green). **(H)** Immunofluorescence assays showed that intralung injection of shNOTCH1 adenovirus significantly reduced F4/80^+^CD206^+^ TAM recruitment (F4/80, far-red; CD206, green) (*p<0.05, **p<0.01).

Tumor tail vein injection assay was used to test cancer cell metastasis *in vivo*. Liposomal clodronate and DAPT combined injection group had lowest metastasis rate, and the metastases node was extremely smaller than any other treatment (Figure 7A). The number of metastases node in liposomal clodronate and DAPT combined group mice was significantly reduced when compared with liposomal clodronate or DAPT treatment group (Figure 7B). Mice were sacrificed and lung was dissected for further fluorescence and histological analysis. Tissue fluorescence assay showed that the fluorescent intensity of dissected tissue was significantly decreased in liposomal clodronate and DAPT combined group than other groups (Figure 7C). Histological analysis revealed that the morphology and histology of metastases was less tight in combined groups, and the abnormal metastasis cell was also reduced (Figure 7D).

Mesenchymal-Epithelial Transition (MET) was also important for tumour cells plant to metastatic sites. To further investigate whether NOTCH1 involved in MET relied and M2 macrophage directly induced tumor cell planting, we intralung injected adenovirus targeted NOTCH1 to inhibit NOTCH1 expression. Result showed that M2 macrophage direct-contact-induced DU145-luc and 22RV1-luc metastases were decreased by intratissue shNOTCH1 adenovirus injection (Figure 7E). The mouse survival rate was also increased in the shNOTCH1 intralung injection group compared with the control group (Figure 7F). E-cadherin and N-cadherin double immunostaining showed that intratissue shNOTCH1 adenovirus injection diminished cancer EMT levels (Figure 7G). Moreover, shNOTCH1 inhibition of lung tissue also decreased F4/80^+^CD206^+^ M2 macrophage infiltration (Figure 7H). These data suggested that NOTCH1 signaling was essential for M2 macrophage-mediated and MET-relied tumour cells plant.

### Increasing CD68/CD206/N1ICD expression in PCa indicated poor prognosis

In PCa patient tissues, CD68^+^/CD206^+^ TAMs were increased in high-grade PCa tissue, as expected (Figure S5A). Furthermore, CD68^+^/CD206^+^ TAMs were abundant in primary tumors, which proved a high tendency for metastasis (Figure S5B). The Cox proportional hazards model also showed that less CD68^+^/CD206^+^ M2 macrophage infiltration was associated with better prognosis (Figure S5C). Kaplan-Meier survival rate analysis showed that CD68^+^/CD206^+^ M2 macrophage infiltration indicated poor patient survival (Figure S5D). Moreover, CD68^+^/CD206^+^ M2 macrophage infiltration positively correlated with adverse clinical outcomes, including depth of invasion (p<0.05), lymph node metastasis (p<0.0001), distant metastasis (p<0.05), Gleason patterns (p<0.0001) and advanced TNM stage (p<0.0001) (Table S1). Thus, these data demonstrated that M2 macrophage infiltration enhanced PCa malignancy behaviour.

**Figure 8:**
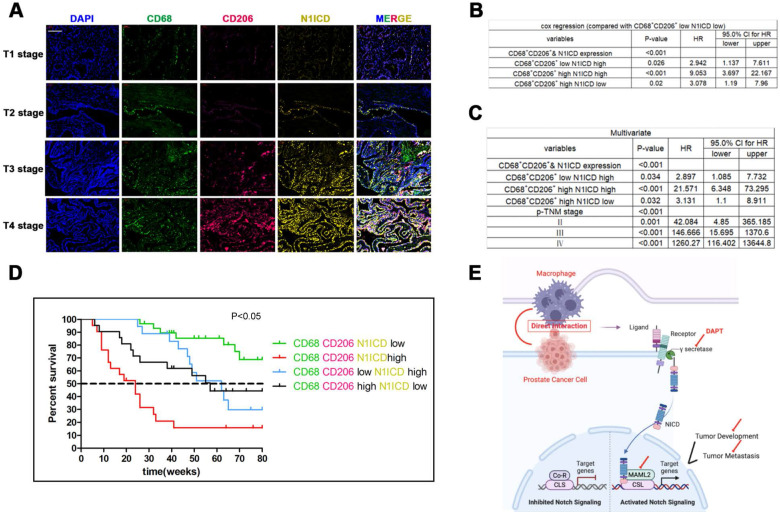
** Recruitment of M2 macrophages in PCa predicts poor prognosis.** Tissue immunofluorescence triple staining of CD68, CD206 and N1ICD revealed that CD68/CD206/N1ICD expression was positively associated with high TNM stage. **(B)** Cox regression analysis showed a significant difference in the CD68/CD206/N1ICD expression status of PCa (p<0.0001). **(C)** Multivariable Cox analysis including prostate cancer-relevant factors (pTNM stage) suggested that CD68/CD206/N1ICD expression was an independent marker for poor prognosis and that upregulation of N1ICD was associated with poor prognosis. **(D)** Kaplan-Meier survival analysis revealed that PCa patients with high CD68/CD206/N1ICD expression had the lowest survival rate. **(E)** Schematic illustration of TAMs directly contacting PCa cells.

Triple immunity staining was used to investigate the clinical value of CD68^+^/CD206^+^ TAMs crosslinked with N1ICD^+^ PCa cells. CD68/CD206^high^N1ICD^high^ was positively associated with high TNM stage (Figure 8A). Univariate Cox analysis showed that high CD68/CD206/N1ICD expression was associated with worse prognosis than other groups (Figure 8B). Multivariable Cox analysis including prostate cancer-relevant factors (pTNM stage) suggested that CD68/CD206/N1ICD coexpression was an independent indicator of poor PCa prognosis (Figure 8C). Furthermore, CD68/CD206^high^N1ICD^high^ PCa patients possessed the lowest survival rate compared with any other group (Figure 8D). An overall illustration of TAMs directly contacting PCa cells is shown in Figure 8E. In conclusion, these data, together with our previous data, preliminarily revealed that increased CD68^+^/CD206^+^ M2 macrophage crosslinking with N1ICD^+^ tumor cells was positively correlated with PCa progression.

## Discussion

Tumor-associated macrophages (TAMs) are one of the most important components in the tumor microenvironment (TME). They mainly originated from circulating monocytes and are recruited to the primary tumor. They programmed by tumour derived environment, such as colony-stimulating factor-1 (CSF-1) and other chemokines, hypoxia, immunity checkpoint inhibitors etc. 21, 22. As mentioned, TAMs polarization is complicated though mostly polarized as M1/M2. M1 macrophages are traditionally treated as anti-tumoral, when M2 have pro-tumoral effect. But this theory is challenged: M1/M2 polarization is an oversimplification which can't show the heterogenous TAMs plasticity. Very recently, evidence showed that M1 macrophages induce pro-tumor inflammation in melanoma cells via TNFR-NF-κB signaling 23. Also fibroblasts in the tumor also could induce trans-differentiation, from anti-tumorigenic macrophages to pro-tumor TAMs 24. Even though, accumulating evidence indicates that TAMs exert cancer progression and immunity treatment resistance 25-28.

The way TAMs interact with tumor cells should be clearly performed in order to treat macrophages as a therapeutic target. Paracrine is well studied as TAMs' great secretion character. Besides, TAMs also could express large amounts of surface ligands and receptors that are utilized to transfer or receive cell signals. The expression patterns of these receptors and their ligands in tumor cells and macrophages facilitate tumor cell and macrophage migration toward each other and together penetrate a dense collagen matrix 29, 30.In our study, we provide new evidences on the communication between TAMs and PCa cells. The direct contact manner greatly promotes PCa cell proliferation and EMT-associated cell invasion. We demonstrated the updated NOTCH1 pathway in PCa cells contributed to direct contact manner phenomenon.

Altered NOTCH1 signaling pathway is positively involved in tumor cell proliferation and cancer metastasis process 15. Importantly, activated NOTCH signalling abides by ligand and receptor binding, which requires cell-cell contact 31. It has been recently identified that the NOTCH pathway is not a simple linear sequence of events, as it can have crosstalk with other pathways to construct an extremely complex network 32. Though AR pathway was crucial for PCa progression and involved in NOTCH signaling activation in PCa cells 19, we did not observe alterations in AR-dependent transcriptional activity after direct coculture with TAMs. Interestingly, we did not observe alterations in NOTCH ligands and receptors after direct coculture. Through our RNA sequencing, we noticed that γ-secretase complex genes were enriched, as well as MAML2, in M2 macrophage contact PCa cells. As mentioned, MAML2 acts as a coactivator of nuclear NICD and facilitates the conversion of the CLS transcriptional complex from a repressor to an activator of the NOTCH target 33. This implied that M2 macrophage contact coculture increased γ-secretase cleavage and MAML2 expression in PCa cells which were both approaches for NOTCH1-dependent PCa progression.

There are some limitations in this study. TAMs are polarized to a continuum of states between M1 and M2 poles *in vivo*. We only focus the M2 polarization type in this study. Specifically, the activation phenotype of IL-4/IL-13-polarized THP-1- or PBMC-derived macrophages was not fully equal to TAMs in the PCa microenvironment although those macrophages could be classified as M2 ones. In clinical studies, we use CD68/CD206 as the TAMs markers which also can't represent all TAMs in the tissues. Moreover, the way direct contact manner modulate NICD release and MAML2 expression in PCa cells may be further explored to explain this deliciated communication.

## Conclusions

The current study supplied a direct contact model for M2 macrophages and PCa cell communication. M2 macrophage direct contact promotes PCa cell proliferation and metastasis by activating NOTCH signaling. In addition, direct contact increased cellular NICD release and MAML2 expression to promote NOTCH pathway transcription in PCa cells. Depleting NOTCH signaling and M2 macrophage recruitment significantly reduced PCa cell proliferation and metastasis *in vivo*. Thus, our findings not only uncover a novel pattern by which TAMs promote PCa progression but also provide a potential microenvironment target for delaying PCa deterioration.

## Supplementary Material

Supplementary figures and table.
