# Supplementary material for: Tumor-associated macrophages in direct contact with prostate cancer cells promote malignant proliferation and metastasis through NOTCH1 pathway
Source: Int J Biol Sci. 2022 Oct 3;18(16):5994–6007. doi: 10.7150/ijbs.73141 (PMC9682538; doi:10.7150/ijbs.73141)
Supplement: Supplementary file 1 — Supplementary figures and table. [file ijbsv18p5994s1.pdf]

Legends Section for Supporting Information

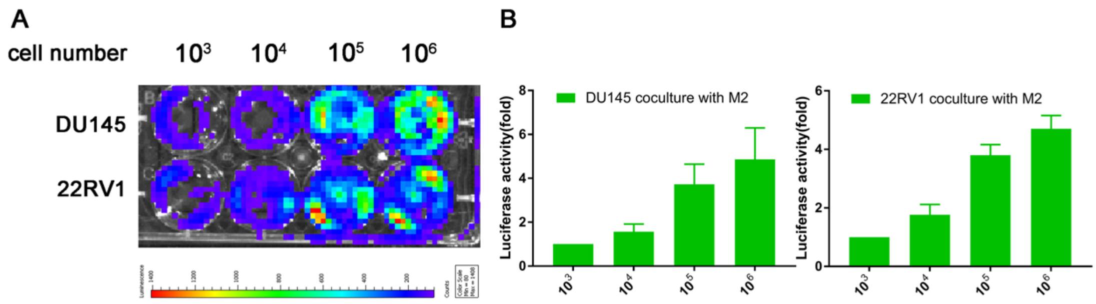

Figure S1 TAMs faciliate prostate cancer malignancy in a M2-dependent manner

（A）The luciferase value was positively corelated with tumor cell number. (B) Quantification data of the intensity value.

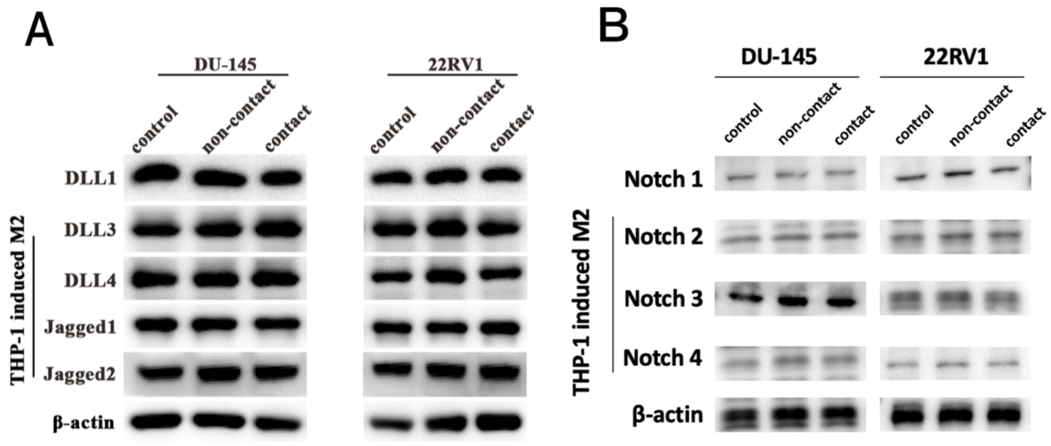

Figure S2 NOTCH signaling ligands and receptors aren't changed in direct contact coculture environment

(A) No expression change was found in ligands (Dll1, Dll3, Dll4 and Jag1, Jag2) in M2 macrophages. (B) No expression change was found in receptors (NOTCH1,2,3,4) in PCa cells.

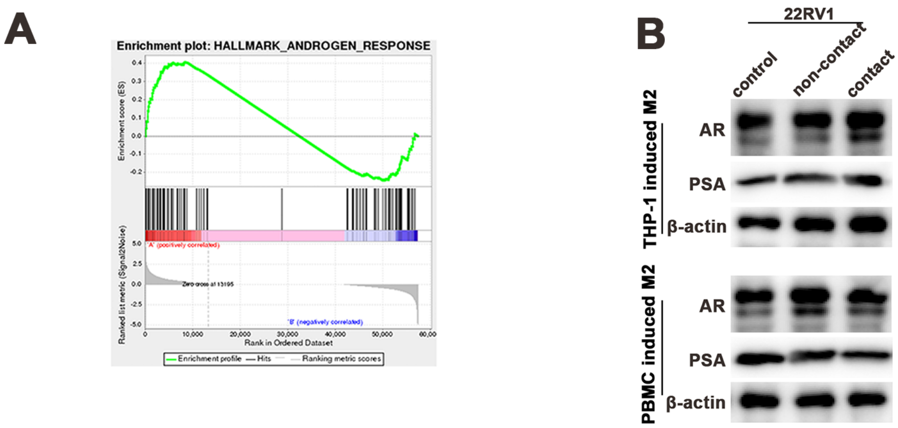

Figure S3 NOTCH signaling activation was not relevant to AR and PSA level in direct contact model

(A) GSEA of AR signaling alteration in 22RV1 cells. (B) The expression of AR and PSA in noncontact and contact cocultured 22RV1 cells was tested by Western blotting.

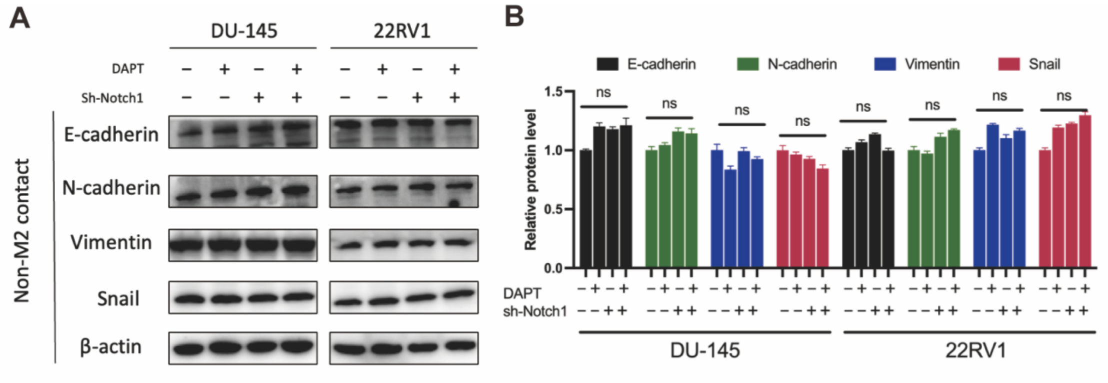

Figure S4 M2 macrophage direct contact coculture other than DAPT or Notch1 depletion promoted EMT process

(A) No expression change of EMT markers was found in DAPT or knockdown Notch1 groups by Western Blot. (B) Quantification data of the intensity value.

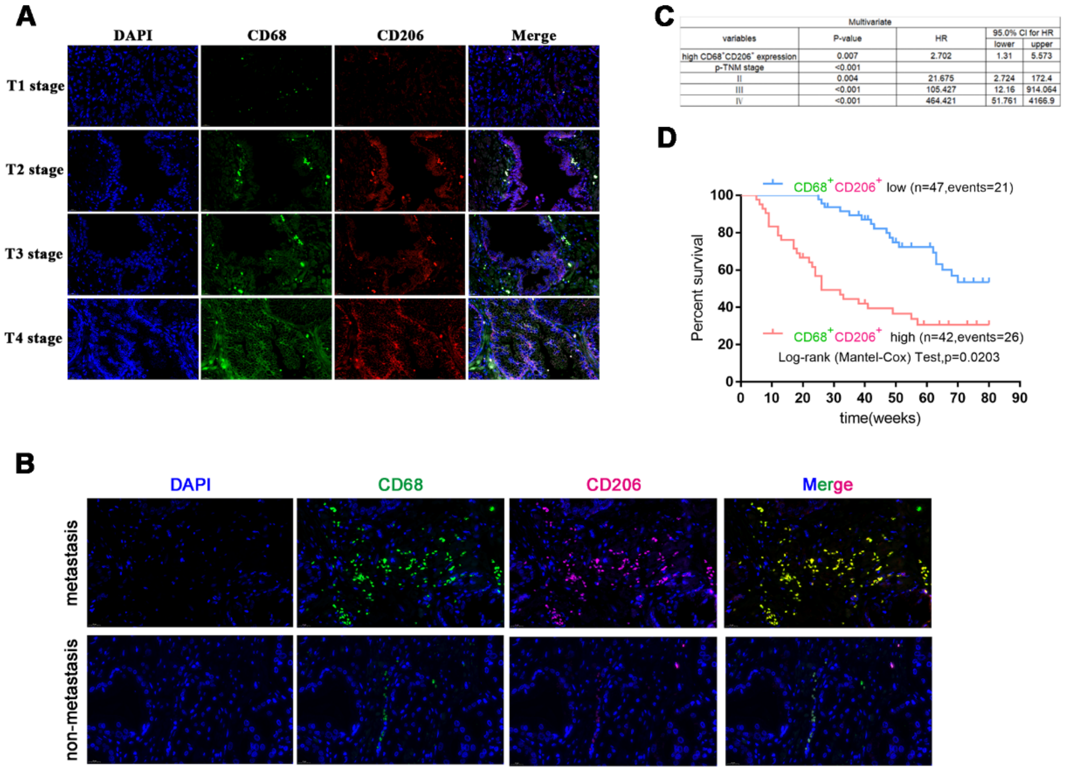

Multivariate				
variables	P-value	HR	95.0% CI for HR	
			lower	upper
high CD68⁺CD206⁺ expression	0.007	2.702	1.31	5.573
p-TNM stage	<0.001			
II	0.004	21.675	2.724	172.4
III	<0.001	105.427	12.16	914.064
IV	<0.001	464.421	51.761	4166.9

Figure S5 M2 macrophage was associated with poorer prognosis and advanced cancer stage in clinical tissues

(A) Tissue immunofluorescence staining of CD68 and CD206 revealed that CD68+CD206+ TAMs were increased in high-grade PCa tissue. (B) IF staining results also showed that CD68+CD206+ TAMs were abundant in primary tumors, which proved a trend toward metastasis according to clinical features. (C) The Cox proportional hazards model also showed that increased CD68+CD206+ TAM infiltration is associated with poor prognosis. (D) The IF staining results of CD68 and CD206 were first quantified, and then Kaplan–Meier analysis was applied, which showed that CD68+CD206+ TAM infiltration is associated with poor patient survival.

Table S1. Association of CD68 and CD206 expression with clinicopathological characteristics of prostate cancer patients

Variables	N	CD68 and CD206 expression (n %)		P value
		Low (≤ 30%)	High (>30 %)	
Age (years)	89	47 (53)	42 (47)	0.562
≤ 60	30	16 (51)	14 (49)	
> 60	59	31 (53)	28 (47)	
Primary Tumor	89	47 (53)	42 (47)	0.018
T1	38	27 (71)	11 (29)	
T2	27	12 (44)	15 (68)	
T3	16	6 (38)	10 (62)	
T4	8	2 (25)	6 (75)	
Regional Lymph	89	47 (53)	42 (47)	< 0.0001
Nodes	69	44 (64)	25 (36)	
N0	20	3 (15)	17 (85)	
N1				
Distant Metastasis	89	47 (53)	42 (47)	0.033
M0	76	44 (58)	32 (42)	
M1	13	3 (23)	10 (77)	
pTNM stage	89	47 (53)	42 (47)	< 0.0001
I	32	21 (66)	11 (34)	
II	19	17 (89)	2 (11)	
III	16	2 (13)	14 (87)	
IV	22	7 (32)	15 (68)	
Gleason Patterns	89	47 (53)	42 (47)	<0.0001
2-5	47	32 (68)	15 (32)	
6-7	22	10 (45)	12 (55)	
8-10	20	5 (25)	15 (75)	

Chi-square test was used for analysis.
